# Fulminant Early-Onset Neonatal Sepsis Due to Streptococcus gallolyticus (Group D): A Report of a Fatal Case

**DOI:** 10.7759/cureus.99903

**Published:** 2025-12-23

**Authors:** Ilham Elouardighi, Lamya El Laziji, Soukaina Batali, Amina Barkat

**Affiliations:** 1 Research Team on Health and Nutrition of Mother and Child, Faculty of Medicine and Pharmacy, Mohammed V University, Rabat, MAR

**Keywords:** fatal, fulminant, group d streptococcus, high-risk newborn, neonatal sepsis

## Abstract

Streptococcus enterococci group D is a rare cause of neonatal sepsis, and it is associated with significant morbidity and a very high fatality rate. The infection is usually acquired intra-partum, from the colonization of the maternal genital tract. Most affected neonates have an early-onset presentation of symptoms, usually within the first 48 hours after birth, which is similar to other causes of neonatal sepsis such as Streptococcus agalactiae or Escherichia coli. We describe an early preterm infant with a fatal, early-onset sepsis caused by Streptococcus D.

## Introduction

Neonatal infections (NIs), both early (occurring in newborns within the first 72 hours of life) and late (occurring in newborns after 72 hours of life), together with prematurity and perinatal asphyxia, represent an important cause of neonatal morbidity and mortality, which may be manifested by direct consequences of the infection or by the inflammatory syndrome they cause. Their incidence for now remains low, less than 10 per 1000 births [1].

“Group D streptococcus” mainly refers to enterococci and gallolytic streptococci; it is a rare cause of neonatal sepsis, associated with significant morbidity and a very high mortality rate. The infection is usually acquired during childbirth, due to colonization of the maternal genital tract.

Currently, the most common cause of neonatal sepsis and meningitis is group B Streptococcus and E. coli [2]. However, group D streptococcus (GDS) is also capable of causing fulminant neonatal sepsis or meningitis that is clinically indistinguishable from that caused by group B streptococcus.

GDS is a rare cause of early neonatal sepsis and neonatal meningitis, with a limited number of cases reported in the literature; hence, there is importance of reporting this clinical case.

We report a case of fulminant early-onset neonatal sepsis with early onset caused by group D streptococcus in a preterm infant in order, to raise awareness of this rare but potentially fatal pathogen in early NIs.

## Case presentation

A newborn boy, born at 34 weeks gestation, from an unmonitored pregnancy, was delivered at Khemisset Provincial Hospital and transferred to the neonatal intensive care unit at Rabat Children's Hospital for respiratory distress from birth; and had a Silverman score of 1 - 2 / 10.

A male newborn from an unmonitored pregnancy was delivered vaginally at the provincial hospital in Khemisset and transferred to the neonatal intensive care unit at the children's hospital in Rabat at eight hours of life for respiratory distress present at birth, with a Silverman score of 1-2/10. The 32-year-old mother had no particular medical history. The Apgar score was 9/10/10 at 1, 5, and 10 min, respectively, and the birth weight was 1950g.

On admission, the newborn was pale, hypotonic, and apyretic at 37°C, with an extended skin recoloration time of 4 seconds, tachycardia at 172 bpm, and normal mean arterial pressure with preserved diuresis. Pleuropulmonary auscultation revealed crackles in both lung fields. Cardiovascular auscultation was unremarkable. The rest of the physical examination was unremarkable except for omphalitis.

**Figure 1:**
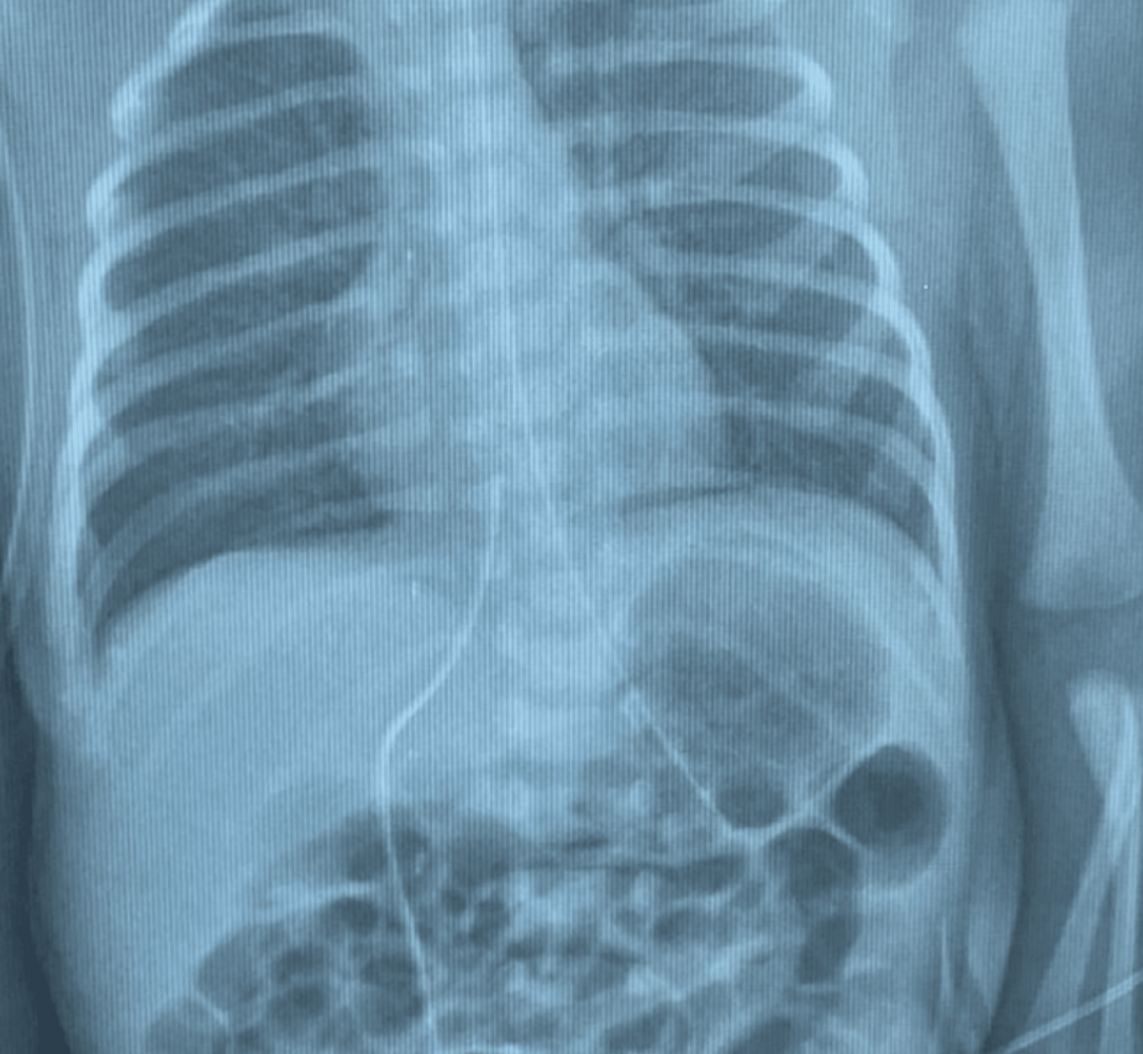
Chest X-ray on admission: some bilateral alveolar-interstitial infiltrates with good lung expansion and proximal air bronchogram

For his respiratory conditions, his blood gas levels showed metabolic acidosis treated with sodium bicarbonate vascular fillers (pH 7.25; pCO2 49 mmHg; HCO3− 21 mmol/L) (Table 1). The admission X-ray showed bilateral pneumonia (Figure 1).

**Table 1:** Laboratory results of biological and bacteriological tests

Parameter	Result	Reference Range	Interpretation
Newborn blood gas analysis	PH 7.25	PH 7.29-7.45	Metabolic acidosis
	PCO2 49 mmHg	PCO2 35-45	
	HCO3- 21mmol/l	HCO3- 24-28	
CRP	214 mg/l	< 6 mg/L	Very high: sepsis
Maternal CRP	51.73mg/l	< 6 mg/L	High
Maternal urine culture	Leukocytes: 39/mm³	Leukocytes:< 10/mm³	Leukocyturia
	Red blood cells: 128/mm³	Red blood cells :< 10/mm³	Hematuria
	GFR glomerular filtration rate: 10^5 ^CFU/ml	GFR glomerular filtration rate: <10⁵ CFU/ml	Positive
Lumbar puncture	Culture: Group D Streptococcus	Culture: negative	Positive for group D streptococcus
	White blood cells: 4/mm^3^	White blood cells: > 10/ml	Normal
	Red blood cells: 100/mm^3^	Red blood cells :> 10/mm^3^	Normal
	Direct examination: negative	Direct examination: negative	Negative
	Culture: sterile	Culture: sterile	Negative
Blood culture	Positive for group D streptococcus	Sterile	Positive for group D streptococcus

The patient was initially placed on non-invasive ventilation with hemodynamic support based on vascular filling and vasoconstrictive drugs and started on a combination of probabilistic antibiotic therapy of a third-generation cephalosporin (ceftriaxone), an aminoglycoside(gentamicin) and penicillin (amoxicillin).

 The infectious history revealed unexplained prematurity at 34 weeks of gestation and a positive maternal infectious assessment: elevated CRP at 51.73 mg/L and a urinary cytobacteriological examination (ECBE) showing leukocyturia of 39/mm³, hematuria of 128/mm³, and a urinary bacterial count of 100,000 CFU/mL. After 24 hours, the culture (on non-selective and selective enriched media) revealed group D streptococcus, isolated and identified using standard techniques, including a latex agglutination test and phenotypic identification on a Vitek 2 automated system. The sensitivity profile, according to the recommendations of the CASFM, demonstrated it to be resistant to amoxicillin, oxacillin, erythromycin, trimethoprim/sulfonamide and doxycycline, but sensitive to amoxicillin/clavulanic acid, third-generation cephalosporin and fluoroquinolones with intermediate sensitivity to aminoglycosides. 

The newborn's laboratory test at 24 hours showed an elevated CRP level of 214 mg/L, a negative lumbar puncture and a positive blood culture for group D streptococcus (S. gallolyticus) (Table 1).

**Figure 2:**
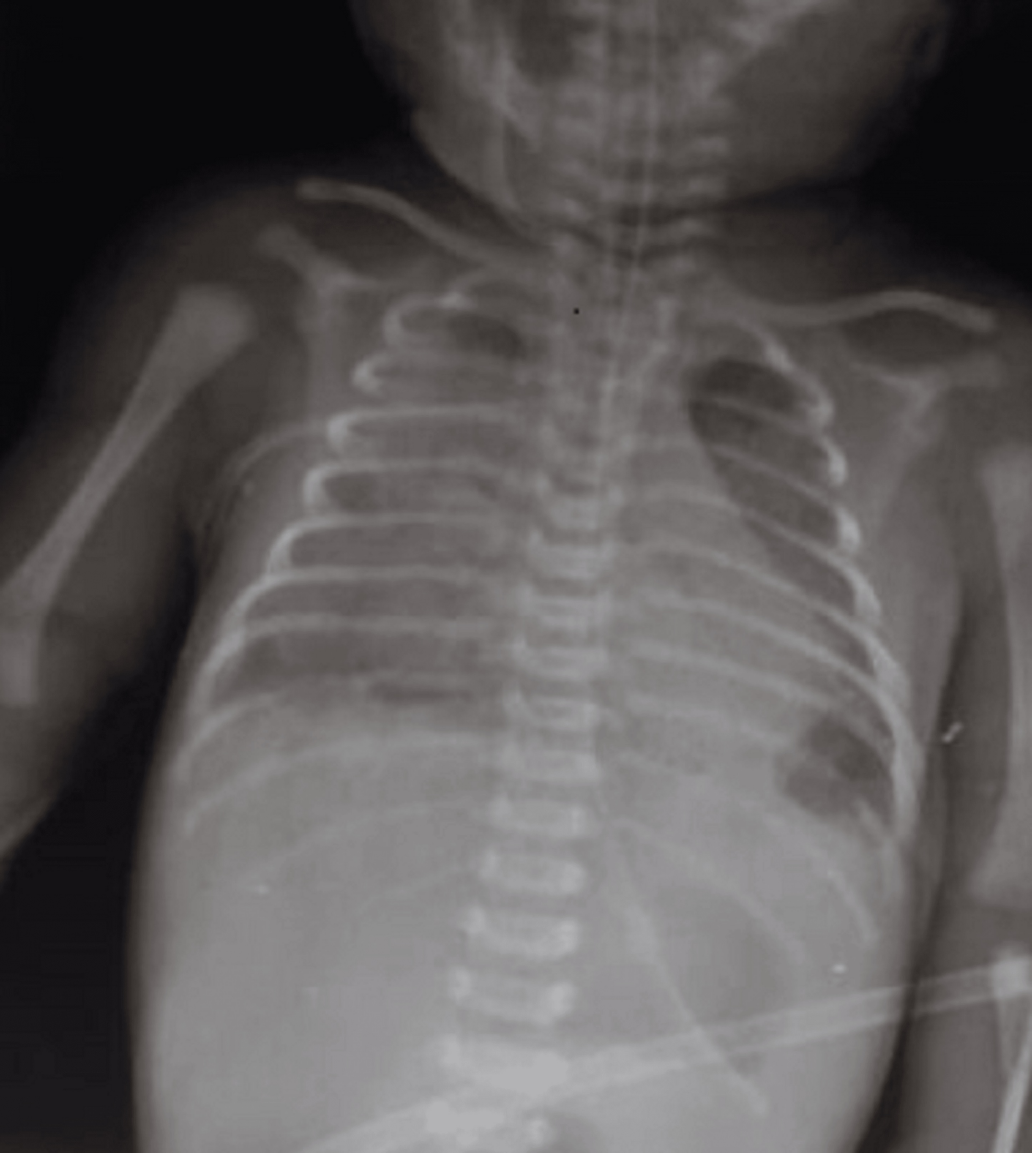
Chest X-ray concomitant with worsening respiratory distress at 48 hours of life showing severe diffuse lung disease (ARDS) Diffuse bilateral infiltrates, loss of lung volume, air bronchograms, consistent with ARDS ARDS: Acute respiratory distress syndrome

The patient's condition deteriorated at 48 hours with worsening respiratory distress and a further deterioration in his hemodynamic status (tachycardia, low mean arterial pressure and oliguria), which required intubation and artificial ventilation with optimization of hemodynamics through further vascular filling and adjustment of vasoconstrictive drug doses. In terms of sepsis, antibiotic therapy was expanded based on the antibiogram to include ceftazidime and amikacin (Table 1). The chest X-ray taken at the same time as the worsening of respiratory distress revealed the presence of severe diffuse pneumopathy causing acute respiratory distress syndrome (ARDS) (Figure 2).

The progression was rapid, with the onset of refractory septic shock and the patient died at 72 hours despite all necessary resuscitation measures.

## Discussion

E. faecalis, E. faecium, E. durans, and E. avium are among the numerous former group D streptococci that have been reclassified and assigned to the genus Enterococcus. For instance, Enterococcus faecalis has replaced Streptococcus faecalis. E. faecium can occasionally be beta hemolytic, and E. faecalis can occasionally be alpha hemolytic [3]. Streptococcus gallolyticus, Streptococcus bovis, and Streptococcus equinus are the remaining non-enterococcal group D strains. Seldom do nonhemolytic streptococci cause disease. Nonhemolytic streptococci should not be confused with weakly hemolytic group D beta-hemolytic streptococci or Listeria monocytogenes, a gram-positive bacillus [4].

Streptococcus D is an unusual pathogen causing early-onset neonatal sepsis. In our case, the signs and symptoms were nonspecific: hypotonia, weak cry, pallor, and dehydration [4,5].

The patient did not have a fever, but examination revealed extreme pallor, generalized hypotonia, and hemodynamic instability. Other causes of sepsis, such as meningitis, were excluded.

Our understanding of the pathophysiology of Enterococcus type D infection in neonates is limited, but it may be associated with perinatal factors. The potential for community transmission from the mother, who may be a carrier, must be considered [5,6].

Early-onset infections can occur from a colonized birth canal or by transplacental hematogenous route as a result of maternal bacteremia [6,7].

An atypical organism, Streptococcus D, can cause sepsis in the first month of life. The patient has similar signs and symptoms found in many patients with hypotonia, pallor, and hemodynamic instability. Fever was absent during the patient's history. Other potential causes of sepsis, such as meningitis, were ruled out.

We do not fully understand how Streptococcus D infects neonatal patients, but many believe the patient's delivery (this is sometimes referred to as the perinatal environment) will be a contributing factor. It is also possible that the mother was a carrier and transmitting from the community may occur during delivery or via the placental blood flow from a mother who has previously had bacteremia, as indicated in our case, the maternal genital tract was colonized [8].

Similar to GBS, S. gallolyticus is often sensitive to penicillin; however, cases with reduced susceptibility to penicillin have been reported,ṅ including two cases of neonatal meningitis due to S. pasteurianus. This organism is also susceptible to aminoglycosides, cephalosporin, and vancomycin, and high rates of resistance to quinolones, macrolides, and tetracyclines have been reported [9].

S. gallolyticus must be considered an important differential for neonatal sepsis, particularly in the presence of hemodynamic instability in the first week of life when maternal streptococcus group B (GBS) is negative. Appropriate identification and classification of the organism are important to further understand the epidemiology of NIs due to S. gallolyticus. Culture sensitivity should be performed to determine the appropriate antibiotic for treatment due to the increasing rates of reduced susceptibility to penicillin [8,9].

Although only one death has been reported in previous cases of neonatal S. gallolyticus infections [1], his case demonstrates that S. gallolyticus can be fatal in low-birth-weight infants, particularly those with extremely low birth weight.

## Conclusions

Although neonatal streptococcus D is infrequent, it must be included as a possible cause of fulminant early sepsis in infants. The association of GDS and neonatal sepsis and meningitis has not been well studied; therefore, much more information is needed to develop effective preventative measures for what could be a deadly infection. Here, we describe the case of a neonate who had early fulminant neonatal sepsis from GDS, which is among the organisms that are very rarely seen as early bacterial infections in newborns. Treatment consists of antibiotic therapy, which must be adjusted based on the results of bacteriological testing, adequate hydration, and appropriate ventilatory and hemodynamic support depending on the patient's clinical condition. Pregnancy monitoring, screening, and prenatal antibiotic prophylaxis (penicillin) for the mother are crucial for prevention.
